# Case report: Novel compound heterozygous missense mutations in the *DDHD2* gene in a Chinese patient associated with spastic paraplegia type 54

**DOI:** 10.3389/fped.2022.997274

**Published:** 2022-08-26

**Authors:** Xin Xu, Fen Lu, Senjie Du, Xiaoke Zhao, Hongying Li, Li Zhang, Jian Tang

**Affiliations:** Department of Rehabilitation, Children's Hospital of Nanjing Medical University, Nanjing, China

**Keywords:** spastic paraplegia, DDHD2, compound heterozygous mutations, intellectual disability, children

## Abstract

**Background:**

Spastic paraplegia type 54 (SPG54) is a rare inherited autosomal recessive disorder, and a complex hereditary spastic paraplegia (HSP) caused by mutations in the phospholipase *DDHD2* gene. SPG54 is characterized by early onset of spastic paraplegia, intellectual disability and dysplasia of corpus callosum.

**Case presentation:**

We report a 9 years and 5 months old Chinese girl with progressive spasm of the lower limbs, muscle weakness and intellectual disability. Brain magnetic resonance imaging (MRI) showed periventricular leukomalacia and thinning of the corpus callosum. According to the Wechsler Intelligence Scale, her IQ is 42. By whole exome sequencing, novel compound heterozygous missense mutations in the *DDHD2* gene [c.168G>C, p.(Trp56Cys) and c.1505T>C, p.(Phe502Ser)] were identified in the proband. Comparative amino acid sequence alignment across different species revealed that Trp56 and Phe502 in the DDHD2 protein were highly conserved during evolution. And multiple *in silico* prediction tools suggested that both mutations were deleterious.

**Conclusions:**

Our study reports a very rare case of complicated HSP caused by two novel compound heterozygous mutations in the *DDHD2* gene. Our findings expand the genetic spectrum of SPG54.

## Introduction

Hereditary spastic paraplegia (HSP) is a group of neurodegenerative monogenic diseases characterized by progressive spasticity and weakness of the lower limbs (1, 2). HSP can be divided into pure subtype and complex subtype according to clinical manifestations. The pure subtype presents only with spastic paraplegia, while the complex subtype has additional neurological symptoms, including intellectual disability, ataxia, optic atrophy, peripheral neuropathy and epilepsy. In addition, HSP shows high genetic heterogeneity. At present, more than 80 monogenic causes have been identified, with inheritance patterns including autosomal recessive, autosomal dominant, X-linked and mitochondrial inheritance (3).

Spastic paraplegia type 54 (SPG54, OMIM: 615033) is a complicated HSP characterized by early onset progressive spasm of lower limbs, accompanied with intellectual disability. Other clinical symptoms include short stature, strabismus, ataxia, optic dysplasia, dysphonia and microcephaly (4). Brain magnetic resonance imaging (MRI) often shows corpus callosum dysplasia and non-specific periventricular white matter lesions. SPG54 is caused by mutations in the *DDHD2* gene (OMIM: 615003) on chromosome 8p11.23 (5). *DDHD2* encodes a phospholipase, which is a member of the intracellular phospholipase A_1_ (iPLA_1_) protein family (DDHD1, DDHD2, and SEC23IP). The DDHD2 protein plays a crucial role in organelle biogenesis and membrane trafficking between the endoplastic reticulum and the Golgi body (6, 7). Here, we report a Chinese case with SPG54 who carried novel compound heterozygous missense mutations in the *DDHD2* gene.

## Case presentation

### Clinical examination

**Figure 1:**
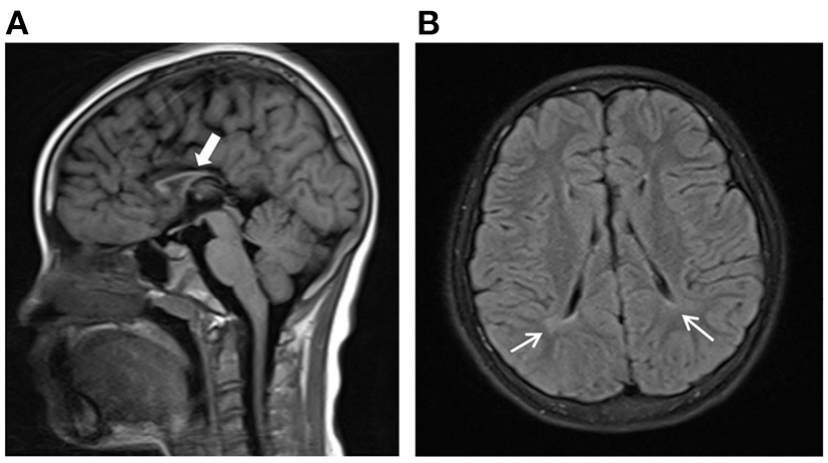
Brain magnetic resonance imaging (MRI) findings of the proband. **(A)** Dysgenesis of the corpus callosum from sagittal view shown on axial T1-weighted images. **(B)** Periventricular white matter hyperintense lesions on FLAIR images.

The proband was a 9 years and 5 months old Chinese girl who was admitted to our Department of Rehabilitation due to abnormal walking posture. She was born at term by spontaneous vaginal delivery and was the first child of healthy and non-consanguineous Chinese parents. She weighed 3 kg at birth and had an Apgar score of 10. She was able to sit unsupported around the age of 8 months and crawl at the age of 1. She can walk independently at the age of 2. But at the age of 4, she gradually showed signs of increased muscle tone and gait impairment. Her most obvious clinical symptom was “toe-walking,” often falling when walking fast. She can't walk long distances and go upstairs as before. And she also had intellectual disability with poor language expression, slow response, attention deficit and poor grades in school. When the girl was admitted to our department for careful examination at the age of 9 years and 5 months, the physical examination revealed a weight of 35 kg (50th to 75th percentile), a height of 128 cm (10th to 25th percentile) and a head circumference of 51 cm (25th to 50th percentile). The girl was non-dysmorphic, and clinically assessed as increased muscle tension of lower limbs, hyperreflexia of the tendons, limited dorsiflexion of both ankles. The results of her blood counts and thyroid profile were normal, as were liver function tests, renal function tests and blood metabolic screen by mass spectrometry. Electroencephalogram (EEG) result was normal. On ophthalmological assessment, there was no sign of optic atrophy. Brain magnetic resonance imaging showed dysgenesis of the corpus callosum (Figure 1A) and periventricular leukomalacia (Figure 1B). The Wechsler Intelligence Scale for Children-Revised (WISC-R) had an IQ of 42, indicating severe intellectual disability. A detailed study of the family history showed that no family members exhibited HSP phenotypes. And no neurological symptom was observed in the parents. They exhibited normal intelligence and walked without abnormal posture. The proband has a 3-year-old sister with normal intelligence and motor development.

### Genetic testing

After approval by the Ethics Committee of Children's Hospital of Nanjing Medical University, 2 mL of peripheral venous blood was extracted from the proband and her family members, including her parents and sister. Genomic DNA was extracted by a DNA extraction kit (Qiagen, Shanghai, China). The DNA library was constructed, and the exomes were captured by xGen^®^ Exome Research Panel v1.0 probe (Integrated Device Technology, USA). The enriched libraries were analyzed on the NovaSeq 6000 Sequencing platform (Illumina, USA). Sequencing reads were mapped to the GRCh37/hg19 reference genome via BWA software. Then candidate genetic variants in exons and canonical splice sites (±2 bp) were picked up with a minor allele frequency <0.005 using the ExAC database (http://exac.broadinstitute.org/), dbSNP (http:// http://gnomad-sg.org/), and 1000 Genomes Project (http://www.1000genomes.org/). Functional prediction and pathogenicity analysis were performed using bioinformatics software. Potential mutations identified by whole-exome sequencing were validated by Sanger sequencing. Suspicious mutations were assessed according to American College of Medical Genetics and Genomics (ACMG) guidelines. Furthermore, we used the online server, ChimeraX (http://www.cgl.ucsf.edu/chimerax//) for analysis to construct the three-dimensional structure of DDHD2.

**Figure 2:**
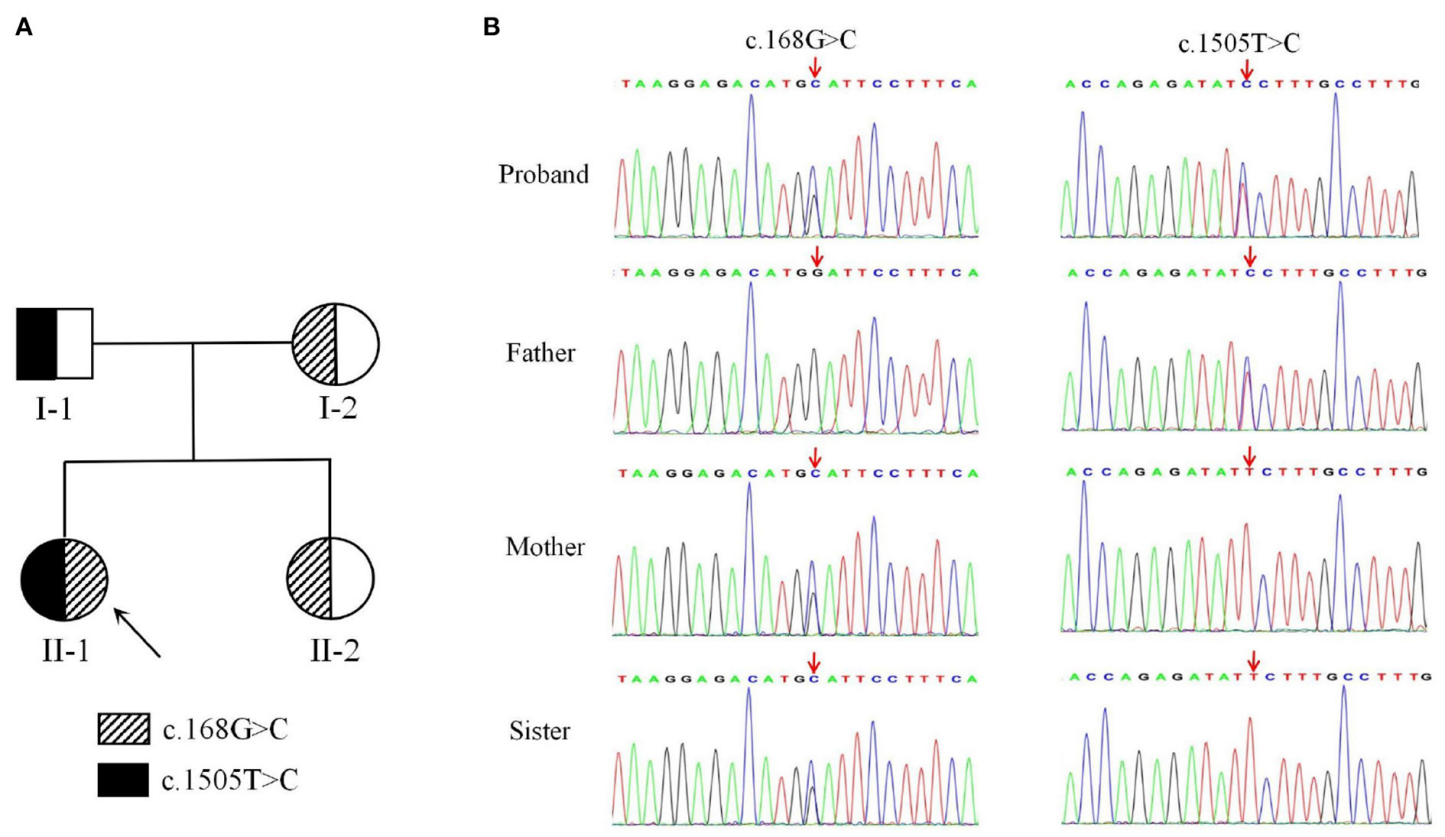
A two-generation family pedigree and Sequencing results of *DDHD2*. **(A)** Heterozygous individuals carrying either mutation are presented with half-filled shaded areas. The arrow indicates the proband. **(B)** Sequencing results showed that c.168G>C (p.Trp56Cys) was inherited from the mother, whereas c.1505T>C (p.Phe502Ser) was inherited from the father.

**Figure 3:**
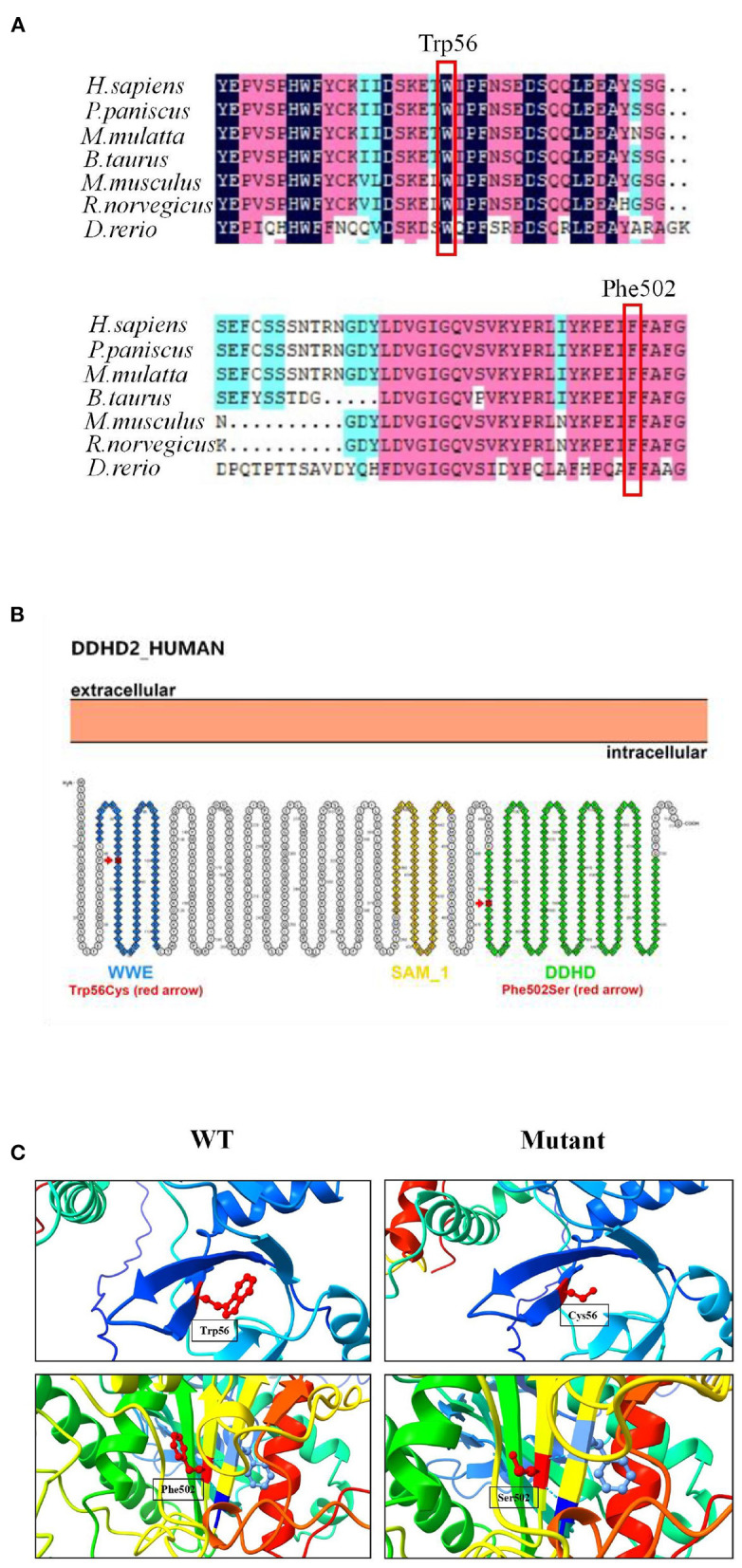
Mutation analysis of *DDHD2*. **(A)** Amino acid sequence alignment of DDHD2 from different species. Mutant amino acids 56 (tryptophan) and 502 (phenylalanine) are highly conserved across species. **(B)** Secondary structure diagram of DDHD2 protein. The p.Trp56Cys mutation located in the WWE domain and the p.Phe502Ser mutation located in the DDHD2 domain. **(C)** Protein molecular models of wild type (WT) and mutant DDHD2 protein (Mutant).

Genetic analysis revealed that the proband had two heterozygous missense mutations in the *DDHD2* gene (Genbank association number: NM_015214), exon 2 c.168G>C (p.Trp56Cys) and exon 13 c.1505T>C (p.Phe502Ser). Sanger sequencing confirmed that her mother and sister carried the c.168G>C (p.Trp56Cys) mutation and her father carried the c.1505T>C (p.Phe502Ser) mutation (Figures 2A,B). Comparative amino acid sequence alignment of DDHD2 across different species revealed that the affected amino acids 56 (tryptophan) and 502 (phenylalanine) are highly conserved (Figure 3A). Furthermore, these two *DDHD2* mutations were not found in ExAC, dbSNP and 1000 Genomes Project databases. The altered amino residues 56 (tryptophan) and 502 (phenylalanine) in the proband are located in the WWE and DDHD domains, respectively (Figure 3B). The two missense changes yielded predominantly deleterious prediction scores by multiple *in silico* prediction tools (SIFT, PolyPhen-2, MutationTaster, Provean and REVEL), and the analysis suggested that these two mutations were predicted to be pathogenic (Table 1).

**Table 1:** Evaluation of the *DDHD2* mutations identified in this study.

***DDHD2* mutation**	**CytoBand**	**Position**	**SIFT score**	**Polyphen-2**	**MutationTaster**	**Provean score**	**REVEL score**	**Novel/reported**
c.168G>C,	8p11.23	38090680	0	1.0	1	−12.34 (< -1.3)	0.729 (>0.5)	Novel
p.Trp56Cys			Damaging	Probably damaging	Disease causing	Deleterious	Deleterious	
c.1505T>C,	8p11.23	38109693	0	0.995	1	−6.88 (< -1.3)	0.757 (>0.5)	Novel
p.Phe502Ser			Damaging	Probably damaging	Disease causing	Deleterious	Deleterious	

Three-dimensional structural modeling of the DDHD2 protein showed that the mutations did not change the hydrogen bonding in protein, but both mutations resulted in changes in side chain size and space ratio compared with the wild type (Figure 3C), which might lead to changes in the conformation of the DDHD2 protein. Videos of the three-dimensional structure of wild type and mutant DDHD2 can be found in the Supplementary material. Based on the above, these two compound heterozygous mutations could be the molecular basis for SPG54 in this case.

## Discussion and conclusion

SPG54 is a rare autosomal recessive neurological disorder characterized by progressive early-onset spasticity, intellectual disability, short stature and dysplasia of corpus callosum. To date, only about 40 patients have been reported all over the world (2–5, 8–18). The phenotypic of our proband is consistent with the characteristic clinical manifestations of SPG54. About 85% of patients with SPG54 develop relevant symptoms before the age of 5. Typical brain MRI manifestations of SPG54 patients are thin corpus callosum and periventricular white matter lesions. Further examination of brain magnetic resonance spectroscopy (MRS) in patients with SPG54 showed abnormal lipid peaks, with the highest intensity around the basal ganglia and thalamus, indicating abnormal lipid accumulation in the brain (11, 12).

The *DDHD2* gene is the only known pathogenic gene for SPG54. It contains 22 exons and encodes a protein composed of 711 amino acids. DDHD2 protein belongs to the phospholipase A1 family. *In vitro* experiments showed that DDHD2 protein has the catalytic activity of phospholipase A1, which can hydrolyze the ester bond on sn-1 with phospholipid acid as substrate. Studies have showed that DDHD2 was a principal triacylglycerol (TAG) lipase in the nervous system as well (19). *DDHD2* is highly expressed in the human central nervous system (CNS), especially in the occipital cortex, cerebellum, and hippocampus. However, the physiological function of DDHD2, particularly in the brain, is not fully understood. The relationship between DDHD2 protein and synaptic transmission and synaptic plasticity has been discovered in recent years, and it has been reported that the loss of DDHD2 function will affect neurocognitive function (19). Inhibition of *DDHD2* expression in the Drosophila CNS resulted in a decrease in the number of presynaptic active areas and the expression of bruchpilot protein (4). Mutations in the *DDHD2* gene can cause SPG54, with spastic weakness of the lower extremities and intellectual disability. Recent studies showed that *DDHD2* knockout (KO) mice develop motor and cognitive impairments and accumulation of lipid droplets in neurons in the brain (20), which was consistent with the abnormal lipid peak in MRS examination of SPG54 patients. The results indicate that *DDHD2* is involved in lipid pathway and plays an important role in the enzymatic metabolism of lipid droplets. In addition, it has been found that *DDHD2* KO mouse embryonic fibroblasts tend to apoptosis, and the loss of DDHD2 function promotes the production of reactive oxygen species (ROS) in mitochondria, thus accelerating cell apoptosis (21). These results may provide clues to the pathogenesis of SPG54.

Until now, approximately 30 *DDHD2* mutations have been reported in Human Gene Mutation Database (HGMD) and literature, among which truncating mutations and missense mutations are the most common. And the information related to the reported *DDHD2* mutations is shown in Table 2. The DDHD2 protein contains WWE domain, GxSxG lipase motif, sterile-alpha-motif (SAM) domain and DDHD2 domain. And most *DDHD2* mutations reported are located in SAM and DDHD2 domains, and a few are located in WWE domain. The missense mutation of the highly conserved DDHD2 domain, p.Asp660His, was reported in five families, which is a hotspot missense mutation (4, 8, 9). Studies have demonstrated that SAM and DDHD2 domains play a significant role in binding phosphoinositol 4-phosphate and affecting phospholipase activity (7). Three missense mutations (p.Trp103Arg, p.Asp660His and p.Val220Phe) have been previously verified *in vitro* experiments, suggesting that the mutant proteins significantly reduce the phospholipase activity (11, 20). In our study, the mother and sister of the proband carried c.168G>C (p.Trp56Cys) mutation in *DDHD2*, and the father carried c.1505T>C (p.Phe502Ser) mutation in *DDHD2*. Compound heterozygous mutations containing both the maternal and paternal mutations were detected in the proband. And these two missense mutations were predicted to be pathogenic by different bioinformatics softwares and have not been reported previously. In this family, only the proband presented SPG54-related clinical phenotypes. The parents and sister were heterozygous carriers of the *DDHD2* gene, but were healthy. Thus, we concluded that the *DDHD2* compound heterozygous mutations were causative mutations for SPG54. However, further experiments are needed to investigate the function of these two mutations *in vitro* or in animal models.

**Table 2:** *DDHD2* mutations associated with SPG54.

**Number**	**Gene**	**Clinical phenotypes**	**Nucleotide change**	**Amino acid change**	**Mutation type**	**References**
1	*DDHD2*	SPG54	c.400C>T	p.Gln134*	Nonsense	Travaglini et al. (2)
2	*DDHD2*	SPG54	1125+1G>T	–	Canonical-splice	Kumar et al. (3)
3	*DDHD2*	SPG54	c.2057delA	p.Glu686Glyfs*35	Frameshift	Schuurs-Hoeijmakers et al. (4) Schuurs-Hoeijmakers et al. (14)
4	*DDHD2*	SPG54	c.1803dupT	p.Thr602Ilefs*18	Frameshift	Schuurs-Hoeijmakers et al. (4)
5	*DDHD2*	SPG54	c.1386dupC	p.Ile463Hisfs*6	Frameshift	Schuurs-Hoeijmakers et al. (4)
6	*DDHD2*	SPG54	c.1546C>T	p.Arg516*	Nonsense	Schuurs-Hoeijmakers et al. (4)
7	*DDHD2*	SPG54	c.859C>T	p.Arg287*	Nonsense	Schuurs-Hoeijmakers et al. (4) Alrayes et al. (13)
8	*DDHD2*	SPG54	c.1978G>C	p.Asp660His	Missense	Schuurs-Hoeijmakers et al. (4) Magariello et al. (8) Citterio et al. (9)
9	*DDHD2*	SPG54	c.1982_1983delAT	p.Tyr661Cysfs*8	Frameshift	Gonzalez et al. (5)
10	*DDHD2*	SPG54	c.307T>C	p.Trp103Arg	Missense	Magariello et al. (8)
11	*DDHD2*	SPG54	c.334C>T	p.Arg112*	Nonsense	Nicita et al. (10)
12	*DDHD2*	SPG54	c.589G>A	p.Gly197Arg	Missense	Nicita et al. (10)
13	*DDHD2*	SPG54	c.2096A>G	p.Tyr699Cys	Missense	Nicita et al. (10)
14	*DDHD2*	SPG54	c.806C>T	p.Pro269Leu	Missense	Nicita et al. (10)
15	*DDHD2*	SPG54	c.942delC	p.Thr314*	Frameshift	Nicita et al. (10)
16	*DDHD2*	SPG54	c.340_342dupACG	–	Inframe	Nicita et al. (10)
17	*DDHD2*	SPG54	c.340dupA	p.Thr114Asnfs*11	Frameshift	Nicita et al. (10)
18	*DDHD2*	SPG54	c.658G>T	p.Val220Phe	Missense	Doi et al. (11)
19	*DDHD2*	SPG54	1057+5C>G	–	Canonical-splice	Novarino et al. (12) Thabet et al. (15)
20	*DDHD2*	SPG54	c.297T>A	p.Tyr99*	Nonsense	Dong et al. (16)
21	*DDHD2*	SPG54	c.335G>A	p.Arg112Gln	Missense	Dong et al. (16)
22	*DDHD2*	SPG54	c.292C>T	p.Arg98Trp	Missense	Salinas et al. (17)
23	*DDHD2*	SPG54	c.759delT	p.Phe253Leufs*13	Frameshift	D'Amore et al. (18)
24	*DDHD2*	SPG54	c.38delA	p.Gln13Argfs*16	Frameshift	D'Amore et al. (18)
25	*DDHD2*	SPG54	c.168G>C	p.Trp56Cys	Missense	Our present study
26	*DDHD2*	SPG54	c.1505T>C	p.Phe502Ser	Missense	Our present study

* means termination codon.

In conclusion, we describe two novel compound heterozygous missense mutations in the *DDHD2* gene in a Chinese patient associated with SPG54. Through whole-exome sequencing and analysis, the newfound missense mutations enrich the *DDHD2* mutation spectrum and provide a genetic basis for clinical diagnosis.

## Data availability statement

The original contributions presented in the study are included in the article/Supplementary material, further inquiries can be directed to the corresponding author/s.

## Ethics statement

The studies involving human participants were reviewed and approved by Ethics Committee of Children's Hospital of Nanjing Medical University. Written informed consent to participate in this study was provided by the participants' legal guardian/next of kin. Written informed consent was obtained from the minor(s)' legal guardian/next of kin for the publication of any potentially identifiable images or data included in this article.

## Author contributions

XX carried out the molecular genetic studies and drafted the manuscript. FL and LZ performed the clinical data. HL and JT assisted with finding some of the research studies. SD and XZ supervised this research and critically reviewed the manuscript. All authors contributed to the article and approved the submitted version.

## Funding

This work was supported by Science and Technology Development Fund of Nanjing Medical University (No. NMUB2020091).

## Conflict of interest

The authors declare that the research was conducted in the absence of any commercial or financial relationships that could be construed as a potential conflict of interest.

## Publisher's note

All claims expressed in this article are solely those of the authors and do not necessarily represent those of their affiliated organizations, or those of the publisher, the editors and the reviewers. Any product that may be evaluated in this article, or claim that may be made by its manufacturer, is not guaranteed or endorsed by the publisher.
