# Supplementary material for: Case report: Novel compound heterozygous missense mutations in the DDHD2 gene in a Chinese patient associated with spastic paraplegia type 54
Source: Front Pediatr. 2022 Aug 26;10:997274. doi: 10.3389/fped.2022.997274 (PMC9458848; doi:10.3389/fped.2022.997274)
Supplement: Supplementary file 1 [file Presentation_1.PPTX]

## Slide 1
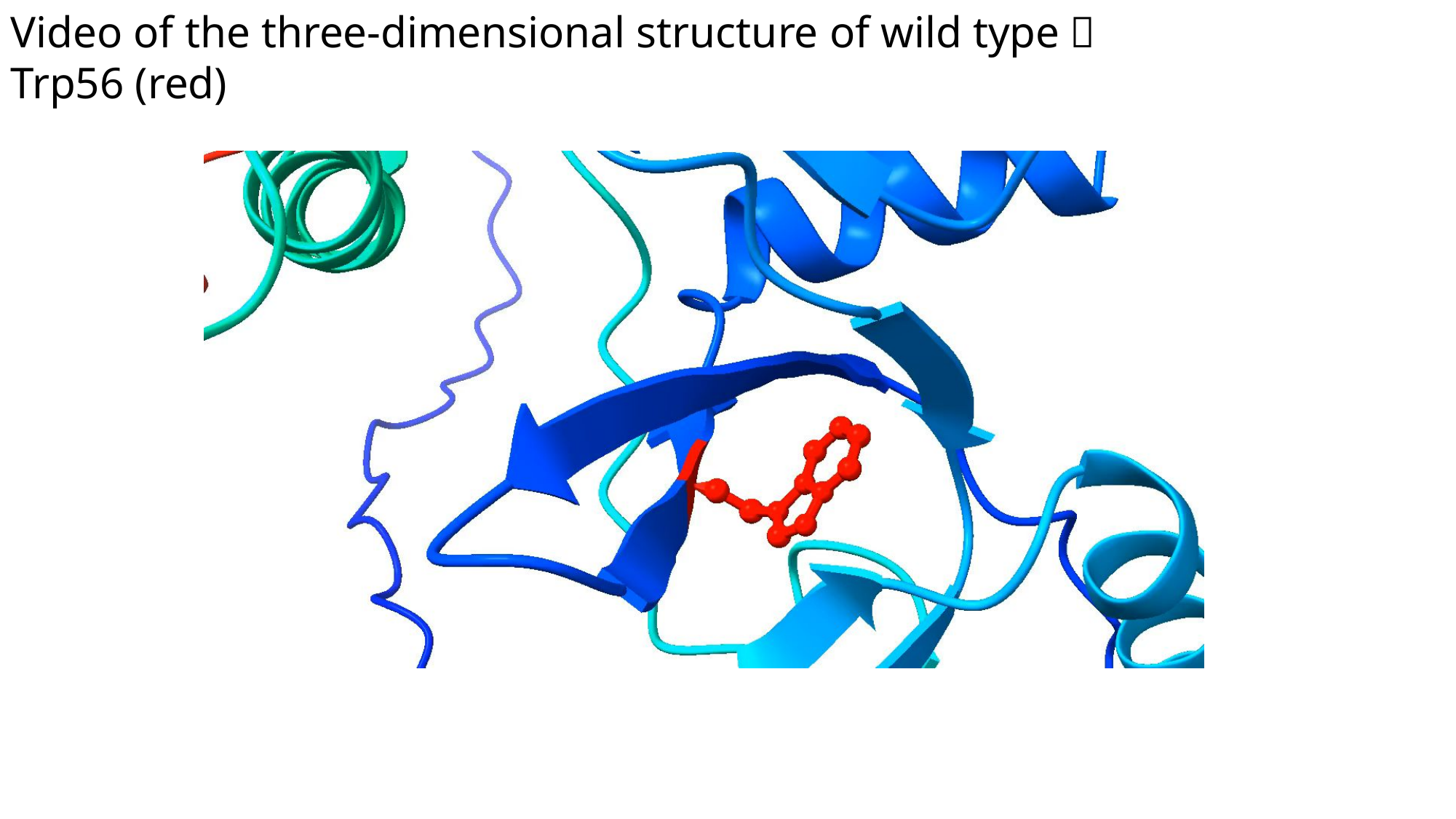

Video of the three-dimensional structure of wild type： Trp56 (red)

## Slide 2
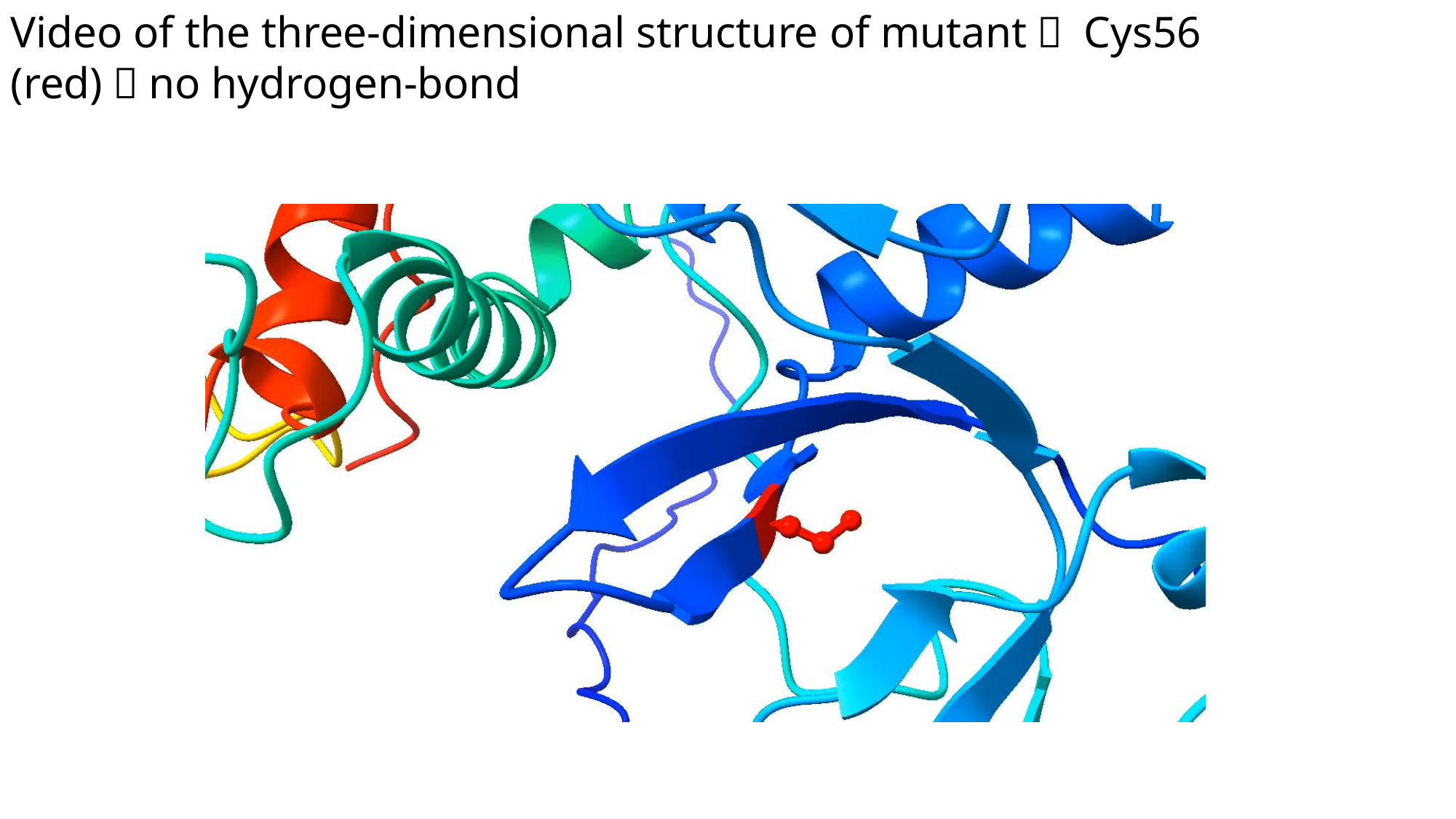

Video of the three-dimensional structure of mutant： Cys56 (red)，no hydrogen-bond

## Slide 3
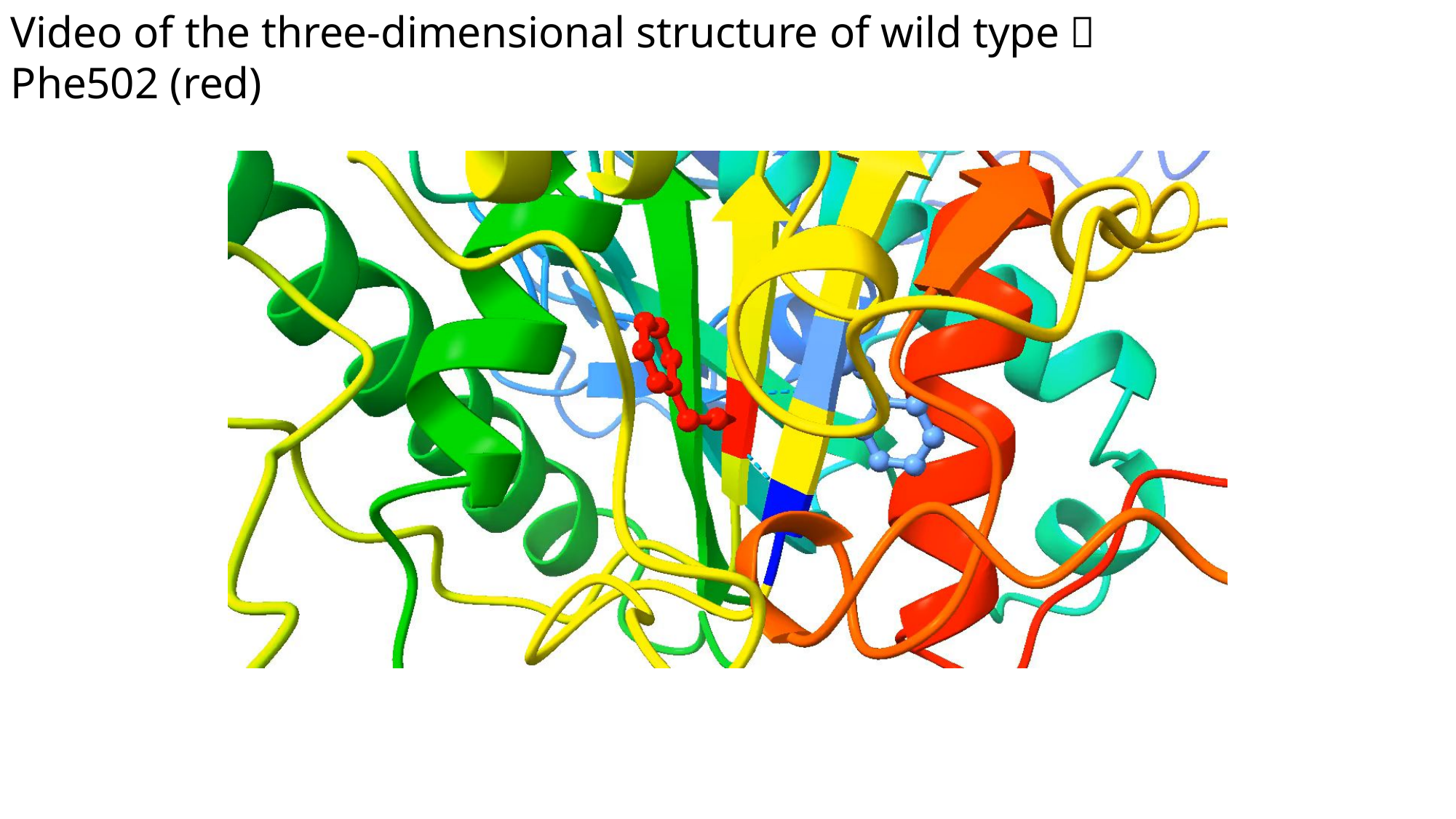

Video of the three-dimensional structure of wild type： Phe502 (red)

## Slide 4
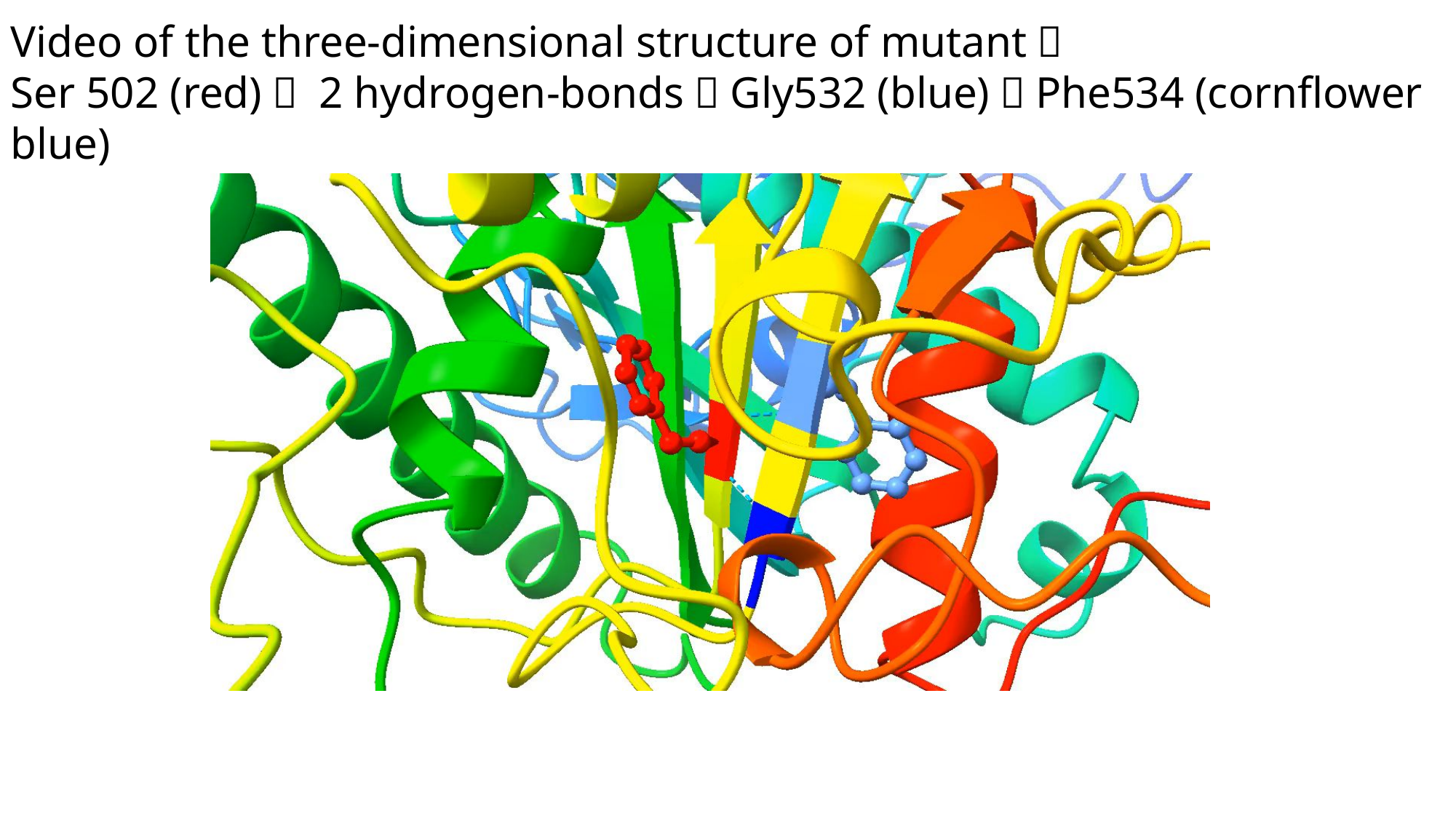

Video of the three-dimensional structure of mutant：
Ser 502 (red)， 2 hydrogen-bonds，Gly532 (blue)，Phe534 (cornflower blue)
